# The CErebro Placental RAtio as indicator for delivery following perception of reduced fetal movements, protocol for an international cluster randomised clinical trial; the CEPRA study

**DOI:** 10.1186/s12884-021-03760-2

**Published:** 2021-04-09

**Authors:** Stefanie E. Damhuis, Wessel Ganzevoort, Ruben G. Duijnhoven, Henk Groen, Sailesh Kumar, Alexander E. P. Heazell, Asma Khalil, Sanne J. Gordijn

**Affiliations:** 1 Department of Obstetrics and Gynaecology, University of Groningen, University Medical Center Groningen, Groningen, The Netherlands; 2 Department of Obstetrics and Gynaecology, Amsterdam University Medical Centers, University of Amsterdam, Amsterdam, The Netherlands; 3 Department of Epidemiology, University of Groningen, University Medical Center Groningen, Groningen, The Netherlands; 4 Department of Obstetrics and Gynaecology, Mater Mothers’ Hospital, Mater Research Institute, University of Queensland, Brisbane, QLD Australia; 5 Maternal and Fetal Health Research Centre, Mary’s Hospital, University of Manchester and Manchester University NHS Foundation Trust, Manchester Academic Health Science Centre, Manchester, UK; 6 Fetal Medicine Unit, St George’s University and St George’s University Hospitals NHS Foundation Trust, London, UK; 7 Vascular Biology Research Centre, Molecular and Clinical Sciences Research Institute, St George’s University of London, London, UK

**Keywords:** Reduced fetal movements, Decreased fetal movements, Cerebroplacental ratio, CPR, Placental insufficiency, Stillbirth, Fetal hypoxia, Neonatal morbidity

## Abstract

**Background:**

Routine assessment in (near) term pregnancy is often inaccurate for the identification of fetuses who are mild to moderately compromised due to placental insufficiency and are at risk of adverse outcomes, especially when fetal size is seemingly within normal range for gestational age. Although biometric measurements and cardiotocography are frequently used, it is known that these techniques have low sensitivity and specificity. In clinical practice this diagnostic uncertainty results in considerable ‘over treatment’ of women with healthy fetuses whilst truly compromised fetuses remain unidentified. The CPR is the ratio of the umbilical artery pulsatility index over the middle cerebral artery pulsatility index. A low CPR reflects fetal redistribution and is thought to be indicative of placental insufficiency independent of actual fetal size, and a marker of adverse outcomes. Its utility as an indicator for delivery in women with reduced fetal movements (RFM) is unknown. The aim of this study is to assess whether expedited delivery of women with RFM identified as high risk on the basis of a low CPR improves neonatal outcomes. Secondary aims include childhood outcomes, maternal obstetric outcomes, and the predictive value of biomarkers for adverse outcomes.

**Methods:**

International multicentre cluster randomised trial of women with singleton pregnancies with RFM at term, randomised to either an open or concealed arm. Only women with an estimated fetal weight ≥ 10th centile, a fetus in cephalic presentation and normal cardiotocograph are eligible and after informed consent the CPR will be measured. Expedited delivery is recommended in women with a low CPR in the open arm. Women in the concealed arm will not have their CPR results revealed and will receive routine clinical care. The intended sample size based on the primary outcome is 2160 patients. The primary outcome is a composite of: stillbirth, neonatal mortality, Apgar score < 7 at 5 min, cord pH < 7.10, emergency delivery for fetal distress, and severe neonatal morbidity.

**Discussion:**

The CEPRA trial will identify whether the CPR is a good indicator for delivery in women with perceived reduced fetal movements.

**Trial registration:**

Dutch trial registry (NTR), trial NL7557. Registered 25 February 2019.

## Background

Maternal perception of reduced fetal movements (RFM) occurs in approximately 6 to 15% of pregnancies [1]. RFM often results from relatively benign causes such as an altered fetal position or maternal distraction due to other activities or stress. In some cases, however, it is an important indicator pointing towards placental insufficiency. Placental insufficiency is usually a chronic process of impaired exchange at the maternal-fetal interface, leading to a deprived nutritional status of the fetus, until acute hypoxia becomes life threatening. If long-lasting and/or severe, fetuses typically become small for gestational age. If the onset of placental insufficiency occurs in late gestation, fetal size is more likely to be within the normal range and the chronic placental dysfunction remains undetected. In these cases, RFM may be the first sign of placental insufficiency and acute fetal compromise [2].

RFM is associated with a 2.4–5-fold increase in stillbirth and other adverse outcomes such as asphyxia, neurodevelopmental impairment in the offspring and maternal hypertensive disease [3, 4]. Although the stillbirth rate (> 28 weeks gestation) in the Netherlands has declined to 2.3 per 1000 in 2015, a large proportion of stillbirths remains unexplained and in 20–30% of cases substandard care is identified. Among these substandard care factors, failure to respond to RFM is important. There is still a large difference in stillbirth rates between high-income countries, varying between 1.4 and 3.7 per 1000 pregnancies from 28 weeks gestational age onwards [5]. Although these rates are declining, there is considerable variation between countries. This variation indicates ample room for improvement.

Stillbirth prediction is difficult and the fear of vital consequences leads to substantial overtreatment of women reporting RFM. Functional parameters, such as Doppler ultrasound and serum biomarkers, can help distinguish the compromised fetuses from healthy fetuses. A low cerebroplacental ratio (CPR) on Doppler ultrasound reflects a decrease in resistance in the middle cerebral artery (cerebral flow) and/or an increased resistance in the umbilical artery (placental flow) indicating redistribution of the fetal circulation: a compensatory adaptation to nutrient and oxygen deprivation due to placental insufficiency. A low CPR can identify a compromised fetus regardless of fetal size [6–9], and using the CPR combined with RFM may aid prediction and consequently prevention of stillbirth [10].

The incidence of an abnormal CPR in RFM is approximately 6% [4]. There are no studies of a management strategy based on an abnormal CPR for RFM. In a systematic review that included pregnancies of any risk profile, the CPR outperformed the umbilical artery pulsatility index in the prediction of emergency delivery for fetal distress and prediction of composite adverse outcome. A pooled sensitivity of 0.93 and specificity of 0.74 for perinatal death and a sensitivity of 0.59 and specificity of 0.91 for a composite adverse outcome was found for a CPR <5th centile (equivalent to CPR < 1.1), independent of birth weight [7, 11]. When analysing individual patient data from studies included in this systematic review, the CPR showed no added predictive value for adverse perinatal outcome beyond the umbilical artery pulsatility index, irrespective of gestational age or fetal size [12]. A possible explanation for this controversy could be that in the latter continuous data were used whereas in the review a cut off value was applied to determine (ab)normality.

It is unclear whether planned delivery for women presenting with RFM based on CPR achieves better outcomes than expectant management. Clinicians need to balance the risks of prolonged placental insufficiency associated with a higher risk of adverse outcomes including death against the risks of immediate delivery [9]. This uncertainty translates into considerable practice variation regarding the use of CPR for management of women reporting RFM [13]. Current guidelines in both the Netherlands and the United Kingdom (UK) for the management of RFM recommend ultrasound for fetal biometry assessment and quantification of amniotic fluid, as well as cardiotocography. Doppler measurements are not recommended in these guidelines other than in research setting [14, 15]. However, in 2017–2018 the CPR was already implemented in 22% of local protocols in the Netherlands and this percentage has probably increased since then [13].

The Lancet Stillbirth series identified the link between RFM and stillbirth as key priority for research [16]. It has been suggested to add other (bio) markers of placental dysfunction to biometry measurements in women reporting RFM [17]. Aside from the Doppler measurements mentioned before, the pulsatility index of the uterine artery is gaining attention as possible marker for placental function. A high uterine artery pulsatility index at term seems to be independently associated with an increased risk of adverse perinatal outcome regardless of fetal size [18]. Furthermore, maternal serum markers for placental function, such as serum soluble fms-like tyrosine kinase-1 (sFlt-1) and placental growth factor (PlGF), have considerable association with adverse outcomes [19, 20]. None of these studies performed subgroup analyses for women reporting RFM. The effect of a societal-awareness-intervention of women reporting RFM was assessed in a large trial including over 400,000 pregnant women. The intervention did not result in a significant reduction of stillbirths, yet did increase iatrogenic (relative preterm) births, suggesting that the solution may not be found in societal awareness but in a better risk assessment at the individual level [21].

The aim of this cluster randomised trial is to compare outcomes in women presenting with RFM at term, randomised to either expectant management or immediate induction of labour based on a low CPR for gestation. We will compare neonatal and maternal outcomes, as well as the healthcare costs in both groups. We will also evaluate the predictive value of serum biomarkers (sFlt-1 and PlGF).

## Methods

### Study objective

To assess whether expedited delivery (induction of delivery within 16 h) in pregnancies at term complicated with RFM and an abnormal CPR (< 1.1) improves neonatal outcome as compared to clinical management where the CPR remains concealed.

### Study design

We will conduct an international cluster randomised controlled clinical trial with randomisation at hospital level to either an open (CPR revealed) or concealed arm, performed in the Netherlands, UK and Australia. The protocol was drafted in accordance with the SPIRIT (Standard Protocol Items: Recommendations for Interventional Trials) statements [22]. The trial was registered at the Dutch Trial Registry (NTR 7557) prior to the start of inclusion, and is embedded within the Dutch Society of Obstetrics and Gynaecology’s (Nederlandse Vereniging van Obstetrie en Gynaecologie) NVOG clinical trial consortium, a collaboration of gynaecology and obstetric departments in the Netherlands.

### Randomisation

Participating hospitals will be randomised to either concealed or revealed CPR results for the duration of the study. Hospitals will be randomised using a computer-generated algorithm, stratified by country and number of deliveries per year.

### CPR concealment

Hospitals randomised to the concealed arm will not base management on the CPR result. The CPR will be performed by a sonographer or other physician who is *not involved* in the participant’s treatment. The measurements made will be printed during the scan and will not be saved and thus not transferred to the data capture software program. Only after delivery the scan results can be completed in the data capture form. This ensures that the CPR result, as well as the pulsatility indices of the umbilical- and middle cerebral artery, are concealed for the treating clinician until after delivery.

### Participants and eligibility criteria

All women with a singleton pregnancy and perceived RFM with a gestational age from 37^+ 0^ up to and including 40^+ 6^ weeks, a fetus in cephalic presentation and normal cardiotocograph are eligible.

Exclusion criteria include:

- Small for gestational age, defined as abdominal circumference < p10 based on the Verburg reference curve [23] and/or estimated fetal weight < p10 according to Hadlock 3 formula [24] on ultrasound;
- Planned caesarean delivery, except an elective repeat caesarean delivery;
- Abnormal (ultrasound) findings that indicate immediate need for delivery*;
- Planned delivery within 4 days of presentation with RFM;
- Major congenital malformations or chromosomal abnormalities that can influence pregnancy outcomes chosen for this study;
- Poorly controlled diabetes mellitus.

*This may for instance mean an absent or reversed end diastolic flow in the umbilical artery, depending on local protocol.

### Outcome measures

Primary outcome is a composite of severe neonatal outcome consisting of: stillbirth, neonatal mortality, Apgar score < 7 at 5 min, pH < 7.10 (umbilical artery), emergency delivery for fetal distress (need for cooling, caesarean section or ventouse/forceps) and severe neonatal morbidity (respiratory distress syndrome, hypoxic ischemic encephalopathy, sepsis, necrotizing enterocolitis and supplementary oxygen therapy (> 4 days)).

Secondary outcomes include:

- Mild and other neonatal outcomes, including hypoglycaemia, hypothermia and admittance to neonatal ward.
- Long-term child outcomes including general health, development and behaviour. Child development and behaviour will be assessed 24 months postpartum using the validated Ages and Stages Questionnaire (ASQ-3) and Child Behaviour Checklist (CBCL/1.5–5).
- Maternal outcomes including health related quality of life, fear of childbirth, and development of hypertensive disorders of pregnancy. Wellbeing and fear and experience of childbirth are assessed using the validated questionnaires European Quality of Life 5-Dimension 5-Level (EQ-5D-5L), Wijma Delivery Expectancy/Experience Questionnaire (WDEQ-A and WDEQ-B) and posttraumatic stress disorder checklist for DSM-5 (PCL-5).
- Analysis of maternal serum markers (PlGF, sFLt-1, and PlGF/sFLt-1 ratio) in the context of normal and abnormal CPR and in relation to (adverse) outcomes and baseline characteristics.
- Analysis of the accuracy of routine placental immunohistochemistry.
- Cost-effectiveness analysis of monitoring-intervention strategy. A short-term and long-term cost-effectiveness analysis from a societal perspective will be performed as well as a budget impact analysis.

### Sample size calculation

We estimate a reduction of the incidence of the composite adverse neonatal outcome from 12 to 8%. The background risk of 12% is extrapolated from the existing literature on RFM as applicable to our population [2, 21, 25–29]. This is an extrapolation because there has been heterogeneity in reported outcome measures in RFM studies. We anticipate an incidence of stillbirth and neonatal mortality (< 28 days) of 0.5%, and an incidence of Apgar score < 7 at 5 min and/or pH < 7.10 umbilical artery and/or emergency delivery for fetal distress of 11% (indicative for peripartum asphyxia). The less common events are grouped into severe neonatal morbidity and include: need for cooling, respiratory distress syndrome, hypoxic ischemic encephalopathy, sepsis, necrotizing enterocolitis and, supplementary oxygen therapy (> 4 days). For severe neonatal morbidity we anticipate an incidence of 1% of which 0.5% overlaps with peripartum asphyxia.

Based on these assumptions, a total of 2160 patients obtained from 24 clusters with 90 patients per cluster (equal numbers of control and intervention), achieve 80.06% power to detect a difference in the primary outcome from 12 to 8%, with an alpha of 5%, and an intra-cluster correlation of 0.002.

### Study setting

Eligible patients are recruited in the Netherlands, UK and Australia. Approximately 24 hospitals will participate, consisting of both academic and large teaching hospitals. Inclusion of a pilot centre in the Netherlands started in June 2020 and overall recruitment in the Netherlands started in January 2021. Recruitment in the UK and Australia is planned to start in the second quarter of 2021. A list of current study sites can be obtained via the website [30].

### Interventions

**Fig. 1:**
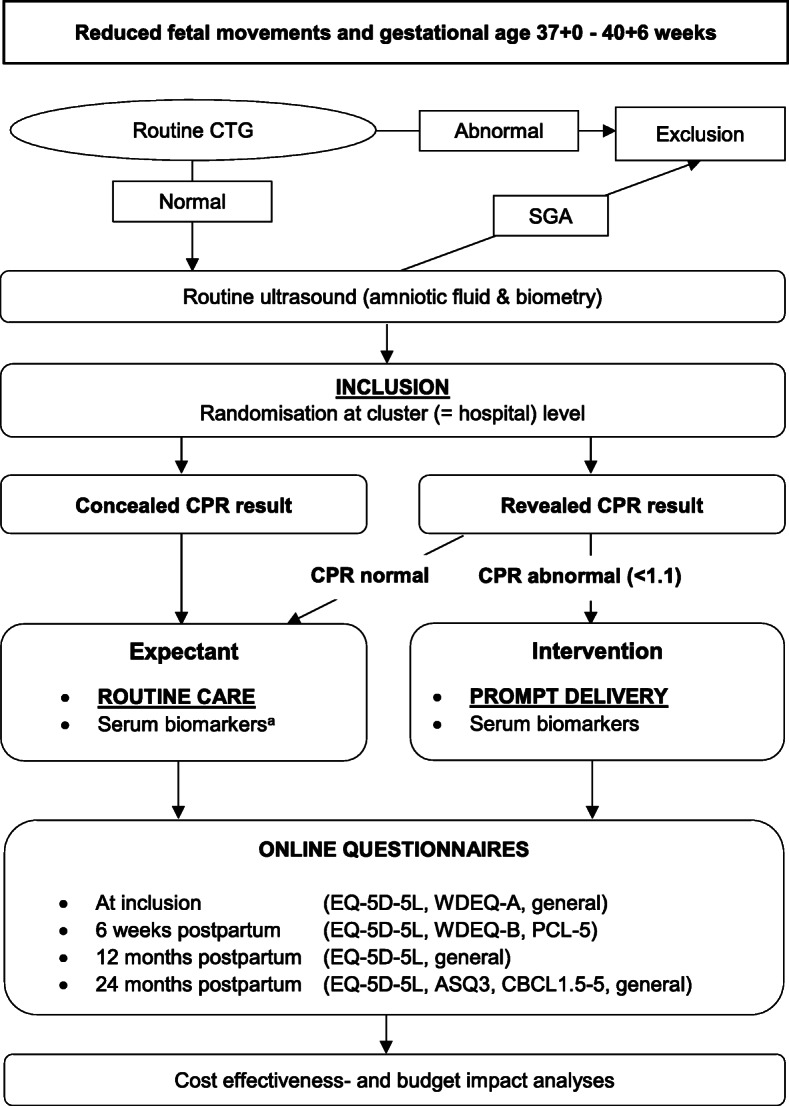
Flow chart of the CEPRA study design and interventions. ^a^ Blood sample will not be drawn from participants with a normal CPR in the open arm. ASQ: Ages and Stages Questionnaire, CBCL: Child Behaviour Checklist, CPR: cerebroplacental ratio, CTG: cardiotocograph, EQ-5D-5L: European Quality of life 5-Dimension 5-Level, PCL-5: posttraumatic stress disorder checklist for DSM-5, SGA: small for gestational age, WDEQ: Wijma Delivery Expectancy/Experience Questionnaire

After recruitment and consent, an ultrasound scan for fetal biometry, amniotic fluid volume, uterine artery pulsatility index and CPR is performed. The pulsatility indices of both the umbilical artery and middle cerebral artery will be reported aside from the calculated CPR. Depending on the cluster, clinicians will be either blinded or unblinded to the CPR. Expedited delivery is pursued in women with an abnormal CPR – defined as an CPR < 1.1 - in the open arm. In this case we aim to start delivery within 16 h or an elective caesarean section will be advanced. There are no criteria for discontinuing the intervention other than participant request. In this case she will be followed-up for intention-to-treat analysis. Women in the concealed arm will not have their CPR results revealed and will receive routine clinical care (Fig. 1).

A maternal blood sample will be taken from *all participants* in the *concealed arm* and from participants with an *abnormal CPR* in the *open arm* for serum biomarker analyses and will be stored locally. Analyses are performed in batch after study closure.

### Additional studies

Depending on local guidelines and feasibility, the placenta is submitted to the local pathology department for routine examination conform the sampling procedures laid out in the Amsterdam Placental Workshop Group Consensus Statement [31]. We aim to analyse the results of routine placental histology performed within routine clinical care for suspected placental insufficiency. Tissue blocks are stored locally and will be analysed centrally for immunohistochemistry and methylation studies after study closure.

### Follow-up

Follow-up consists of online questionnaires at 6 weeks-, 12 months- and 24 months postpartum (Fig. 1).

### Insurance

According to national legal requirements an insurance is taken out that covers injuries caused by participation of this study. The insurance applies to injuries that become apparent during the study period or within 4 years after trial closure. Also a liability insurance in compliance with national legal requirements is taken out.

## Statistical analysis

### Data analysis plan

The primary analysis will estimate the relative risk (RR) with 95% confidence intervals and *p*-values for the composite perinatal outcomes, using a generalized linear mixed model with log link and binomial distribution, with random intercepts and random slopes per cluster. The primary analysis will be performed according to the intention-to-treat principle. A per protocol analyses will be done as secondary analysis.

Dichotomous secondary outcomes will be analysed using the same method as the primary outcome. Continuous data of secondary outcomes will be assessed using a linear mixed model to estimate mean differences, with random intercepts and slopes as appropriate. Median differences will be calculated as appropriate. A significance level of 5% will be used for all tests. Interim testing for efficacy will not be performed, hence the significance level does not need to be lowered.

Given that all required data is collected for routine patient care in both primary and secondary care, missing data is expected to be limited. Multiple imputation will be used if required.

Data of quality of life measures will be obtained at four time points and will be analysed using generalised estimating equations.

For subgroup analyses we will assess the relationship between pregnancy characteristics, outcomes and abnormal serum biomarkers of PlGF and sFlt-1.

### Economic evaluation

A cost-effectiveness analysis (CEA) will be performed based on the empirical data we will obtain in the trial. The CEA will be performed from both a healthcare- and societal perspective. The primary outcome in the short-term CEA will be the composite neonatal outcome. Costs will include pregnancy monitoring, delivery according to place of birth (hospital or at home), mode of delivery and postnatal care. Additional analyses will be performed to determine the effect of maternal biomarker-based risk selection (normal or abnormal PlGF) on cost-effectiveness.

The long-term economic impact and CEA will be evaluated in a scenario analysis comparing costs of CPR monitoring to costs of current routine monitoring, including long-term costs caused by poor neonatal outcome such as physical and intellectual disability. The cost difference will be related to the difference in life-years for the newborns to reflect the effect of avoided stillbirth.

The budget impact analysis of management based on CPR in women with RFM will be performed according to the ISPOR guidelines from a societal perspective as well from the health insurance and/or Dutch healthcare budgeting framework perspective [32]. Also the incremental cost effectiveness ratio will be calculated by dividing the difference in costs between expectant management and the intervention by the difference in quality-adjusted life years between usual care and intervention.

## Ethics and disemmination

### Patient consent

Eligible patients obtain verbal and written information when the CTG appears normal. The information can be read during the CTG recording and a short animation video that has been developed to provide a study summary in laymen’s terms can be watched. If a patient agrees to participate, written informed consent will be obtained by an authorized person. Additional consent will be obtained to store the residual material of the maternal blood sample in a biobank for 50 years and to be approached for follow-up- and future research.

### Data collection and confidentiality

Data will be collected via Castor Electronic Data Capture, a web-based electronic case record form that meets Good Clinical Practice (GCP) guidelines. All data will be handled confidentially and access to the key of the security code is strictly limited. After termination of the study the research data will be preserved for 15 years in keeping with the General Data Protection Regulation.

Participants may withdraw at any time for any reason if they wish to do so. Unless they refuse to allow further data collection, trial data will be collected for the intention-to-treat analysis. If a participant refuses the study intervention for any reason, follow-up (questionnaires) will be continued.

Data management procedures and data collection forms can be found on the website [30].

### Monitoring and safety

An independent data monitoring committee will monitor data capture in compliance with GCP and other (inter) national rules and regulations in order to achieve high quality research and secure patient safety. Each participating center will have a monitor visit at the start- and end of the study and during the recruitment period. Serious adverse events and any other significant problems will be reported to the ethics committee of the University Medical Center of Groningen. An independent Data Safety Monitoring Board (DSMB) has been established. The DSMB will review outcome data of the first 600 and 1200 patients. An interim analysis for efficacy will not be performed. The DSMB can recommend early termination of the study in case of serious safety concerns and a perceived negative benefit-risk balance. These agreements have been documented in a DSMB charter which can be found on the website [30].

### Ethics

The trial will be conducted in compliance with the contemporary version of the Declaration of Helsinki, the ICH GCP and other applicable regulations. Ethical approval was obtained from the ethical board of the University Medical Center of Groningen, the Netherlands (METc 2019.488) and from Mater Misericordiae Ltd. Human Research Ethics Committee, Australia (HREC/MML/65382). The ethics approval from the UK is pending, with recruitment planned to begin once approval has been granted.

### Dissemination plan

Dispersion of the trial results will be accomplished by publication in an international peer-reviewed scientific journal and by presentations at (inter) national conferences. When the results of the trial warrant changes in the standard treatment guidelines of RFM, we anticipate that the widespread execution of the trial in sites throughout the Netherlands, the UK and Australia will aid in its implementation.

## Discusson

The lack of diagnostic tools to correctly identify the fetuses at risk for adverse outcomes and imminent stillbirth as a result of placental insufficiency within the large group of pregnancies reporting RFM, is an everyday problem for midwives and obstetricians. The CPR is a promising functional marker to detect placental insufficiency and is increasingly implemented in clinical practice. Large practice variation in the use of Doppler measurements for the indication of RFM exists among both maternity hospitals and healthcare professionals. The utility of the CPR on which to base clinical management and time delivery in women with perceived RFM has not been investigated. It is possible that expedited delivery does not reduce the adverse neonatal outcomes. Furthermore, other functional markers (including serum biomarkers and the component pulsatility indices of the umbilical artery, middle cerebral artery and uterine artery) have not been assessed for RFM and risk calculation. Evidence based guidelines on the use of Doppler assessment for the indication of RFM are needed.

### Strengths and challenges

A strength of this proposed study is the large control group. The concealed arm (control group) is, however, a logistic challenge. Several options to conceal the CPR result have been explored. Concealing the CPR result at software- or ultrasound device level appeared to have many organisational (many systems available for both ultrasound machines and ultrasound data capture software) and legal limitations. Despite considerable efforts by ultrasound companies and ultrasound data capture software builders, this blinding method proved to be unfeasible. The current approach in which the CPR is concealed for the treating physician was considered to be a proper alternative and is extensively described in the methods section.

Another strength is the sampling of serum biomarkers. The predictive value of maternal biomarkers to detect placental insufficiency at term is currently unknown. In combination with the obtained Doppler results we hope to facilitate the first steps in developing an individualised prediction model to detect pregnancies at risk for placental insufficiency.

The cluster design was preferred over randomisation at the individual patient level because it was expected that both clinicians and the pregnant women would not be willing to wait for spontaneous delivery once an abnormal CPR is measured. Randomisation at cluster level was postulated to lead to a better implementation of the intervention in participating sites.

This cluster RCT will determine whether the CPR is an effective marker to guide management in pregnancies at term complicated with RFM. If the CPR proves to be an adequate indicator for delivery, expedited delivery will be restricted to the smaller number of compromised fetuses suffering from placental insufficiency, reducing their stillbirth risk and improving long-term health. Moreover, a larger group of women with perceived RFM can be reassured and monitored less intensively, reducing their burden of unnecessary intensified monitoring and interventions, resulting in substantial lowering of health costs.

## Data Availability

The datasets used and/or analysed during the current study will be available from the corresponding author on reasonable request.

## Abbreviations

ASQ: Ages and Stages Questionnaire
CBCL: Child Behaviour Checklist
CEA: Cost-effectiveness Analysis
CPR: Cerebroplacental Ratio
DSMB: Data Safety Monitoring Board
EQ-5D-5L: European Quality of life 5-Dimension 5-Level
GCP: Good Clinical Practice
ISPOR: International Society for Pharmacoeconomics and Outcomes Research
METc: Medical research ethics committee
NTR: Dutch trial registry
PCL-5: Posttraumatic stress disorder Checklist for DSM-5
PlGF: Placental Growth Factor
RFM: Reduced fetal movements
RR: Relative Risk
SPIRIT: Standard Protocol Items Recommendations for Interventional Trials
sFlt-1: Soluble fms-like tyrosine kinase-1
UK: United Kingdom
WDEQ: Wijma Delivery Expectancy/Experience Questionnaire
